# Local expression of matrix metalloproteinases, cathepsins, and their inhibitors during the development of murine antigen-induced arthritis

**DOI:** 10.1186/ar1466

**Published:** 2004-12-10

**Authors:** Uta Schurigt, Nadine Stopfel, Marion Hückel, Christina Pfirschke, Bernd Wiederanders, Rolf Bräuer

**Affiliations:** 1 Institute of Pathology, Friedrich Schiller University, Jena, Germany; 2 Institute of Biochemistry I, Friedrich Schiller University, Jena, Germany

**Keywords:** Affymetrix oligonucleotide chips, cathepsins, cytokines, matrix metalloproteinases, murine antigen-induced arthritis

## Abstract

Cartilage and bone degradation, observed in human rheumatoid arthritis (RA), are caused by aberrant expression of proteinases, resulting in an imbalance of these degrading enzymes and their inhibitors. However, the role of the individual proteinases in the pathogenesis of degradation is not yet completely understood. Murine antigen-induced arthritis (AIA) is a well-established animal model of RA. We investigated the time profiles of expression of matrix metalloproteinase (MMP), cathepsins, tissue inhibitors of matrix metalloproteinases (TIMP) and cystatins in AIA. For primary screening, we revealed the expression profile with Affymetrix oligonucleotide chips. Real-time polymerase chain reaction (PCR) analyses were performed for the validation of array results, for tests of more RNA samples and for the completion of the time profile. For the analyses at the protein level, we used an MMP fluorescence activity assay and zymography. By a combination of oligonucleotide chips, real-time PCR and zymography, we showed differential expressions of several MMPs, cathepsins and proteinase inhibitors in the course of AIA. The strongest dysregulation was observed on days 1 and 3 in the acute phase. Proteoglycan loss analysed by safranin O staining was also strongest on days 1 and 3. Expression of most of the proteinases followed the expression of pro-inflammatory cytokines. TIMP-3 showed an expression profile similar to that of anti-inflammatory interleukin-4. The present study indicates that MMPs and cathepsins are important in AIA and contribute to the degradation of cartilage and bone.

## Introduction

Rheumatoid arthritis (RA) is a chronic destructive autoimmune disease characterized by the inflammation and progressive destruction of distal joints. The initial histological features of RA are characterized by synovial lining hyperplasia, excessive angiogenesis and the accumulation of polymorphonuclear and mononuclear cells in the synovium [1,2].

The etiology of RA is still unknown, but the degradation of cartilage and bone observed in RA is caused by an increased expression of proteinases, resulting in an imbalance of these degrading enzymes and their inhibitors [3,4]. Proteinases have a pivotal function in endogenous angiogenesis, antigen presentation and pathological remodeling of cartilage and bone [5-7]. For an understanding of the pathogenesis of RA, it is important to investigate the time profiles of expression of proteinases and proteinase inhibitors during the development of arthritis and their relationship to cytokine expression.

It has been suggested that the immune hyper-responsiveness in RA tissues is triggered by an unknown joint-specific antigen. Antigen-induced arthritis (AIA) in mice is an experimental model for RA, in which arthritis is induced by systemic immunization with the antigen methylated bovine serum albumin (mBSA) in complete Freund's adjuvant, followed by a single intra-articular injection of the antigen into the knee joint cavity [8]. The development of chronic arthritis is visible for several weeks. The advantage of AIA over other experimental arthritis models consists in the exactly defined stages of the development of arthritis elicited by antigen injection into the knee joint cavity. After this initiation of AIA, it is possible to distinguish between the acute stage from day 0 to day 7, characterized by inflammatory processes, and the subsequent chronic stage with pannus formation and joint destruction.

As in RA, breakdown of articular cartilage and bone in AIA results from the overexpression of proteinases and deregulation of the balance between proteinases and their inhibitors. We investigated the expression patterns of matrix metalloproteinases (MMPs), tissue inhibitors of matrix metalloproteinases (TIMPs), cathepsins and cystatins by Affymetrix oligonucleotide microarray technology in combination with real-time polymerase chain reaction (PCR), to identify the mediators that were differentially expressed in murine AIA. We were able to follow the expression in murine knee joints from the induction of arthritis to the development of the chronic phase. Additionally, we studied the expression profiles of different cytokines on mRNA and protein level. To complete our oligonucleotide chip and PCR results, we investigated MMP expression and activity by fluorescence assays and zymography.

## Materials and methods

### Animals

Female C57Bl/6 mice (age 7–9 weeks) were obtained from the Animal Research Facility of Friedrich Schiller University, Jena, Germany, and Charles River Laboratories, Sulzfeld, Germany, respectively. They were kept under standard conditions in a 12 hours:12 hours light:dark cycle and fed with standard pellets (Altromin no. 1326, Lage, Germany) and water *ad libitum*. All animal studies were approved by the governmental committee for animal protection.

### Immunization and arthritis induction

Mice were immunized on days – 21 and – 14 by subcutaneous injections of 100 μg of mBSA (Sigma, Deisenhofen, Germany) in 50 μl of saline solution, emulsified in 50 μl of complete Freund's adjuvant (Sigma), adjusted to a concentration of 2 mg/ml heat-killed *Mycobacterium tuberculosis *strain H37RA (Difco, Detroit, MI, USA). In addition, intraperitoneal injections of 2 × 10^9 ^heat-killed *Bordetella pertussis *bacteria (Chiron Behring, Marburg, Germany) were performed on the same days. Arthritis was induced on day 0 by injection of 100μg of mBSA in 25 μl of saline solution into the right knee joint cavity.

### Joint swelling

For clinical monitoring of AIA development, the joint diameters were analyzed before (nonarthritic mice, immunized with mBSA: day 0) and at various times after AIA induction (days 1, 3, 7, 14 and 21). The joint swelling was measured with an Oditest vernier caliper (Kroeplin Längenmesstechnik, Schluechtern, Germany). Joint swelling was expressed as the difference in diameter (mm) between the right knee joint on days 1, 3, 7, 14 and 21, and the same knee joint on day 0 before arthritis induction. For measurement of joint swelling, the mice were anesthetized by ether inhalation.

### Preparation of total RNA

The expression of mRNA for proteinases and cytokines was analyzed for each individual animal. Arthritic and control mice were anesthetized with ether and killed by cervical dislocation. Then right knee joints (where arthritis was induced) were dissected and skinned. The muscle tissue was removed and the bony parts of knee joints were prepared, including the joint capsules with synovial tissue. The RNA in the knee joint was stabilized in RNAlater (Qiagen, Hilden, Germany), in accordance with the instructions in the manual. After incubation of the joints for 12 hours at 4°C in RNAlater, the samples were transferred to -80°C. The joints were mechanically disrupted by milling with a dismembrator U (Braun AG, Meiningen, Germany) for 15 s at 2000 Hz, followed by cooling the vessel in liquid nitrogen for 1 min. This procedure was repeated six or seven times. The tissue powder was rapidly transferred into 2 ml of cold TRIzol (Invitrogen, Carlsbad, CA, USA), immediately followed by dispersion for 1 min with a Polytron 1200 CL (Kinematica AG, Littau/Luzern, Switzerland). After homogenization and mechanical disruption, the RNA was extracted with TRIzol in accordance with the manual.

### Microarray data analysis

The MG_U74Av2 oligonucleotide chip (array A; Affymetrix, Santa Clara, CA, USA), representing all functionally characterized sequences (about 6000 genes) in the Mouse UniGene database (Build 74) and additionally about 6000 expressed sequence tag clusters, was used to analyze the gene expression in murine arthritic knee joints during AIA.

We hybridized six chips with samples from two individual animals from each investigated time point (two at day 0 [control], two at day 3 [acute phase] and two at day 14 [chronic phase]). RNA labelling, hybridization and scanning of gene chips were performed in accordance with the supplier's instruction. Expression levels were calculated with the commercially available software MAS 5.0 provided by Affymetrix. Normalization of the signal was based on the expression of the housekeeping gene β-actin. Expression in the acute and chronic inflamed knee joints was compared with expression in the knee joints of control animals at day 0.

### DNase treatment and complementary DNA (cDNA) synthesis for real-time PCR

The DNase treatment was performed by DNA Free™ kit (Ambion, Woodward, Austin, TX, USA) in accordance with the manufacturer's instructions. Total RNA (5 μg) was digested with 1 μl of DNase I (1 U/μl) and 2 μl of 10 × DNase I buffer in a volume of 20 μl. Supernatant (15 μl), containing the DNase-treated RNA, was transferred into a fresh 0.5 ml PCR tube. The RNA was denatured by incubation at 65°C for 15 min. After incubation on ice for 5 min, the reverse transcriptase (RT) reaction was completed by adding 35 μl of RT reaction mix, containing 1 μl of Superscript RT (200 U/μl; Invitrogen), 10μl of 5 × RT buffer (Invitrogen), 5 μl of 0.1 M dithiothreitol (Invitrogen), 5 μl of dNTPs (10 mM; Promega, Mannheim, Germany), 2 μl of poly(T) primer (T_30_VN; 50μmol), and 12 μl of distilled water.

Reverse transcription was performed at 42°C for 1 hour, after which the cDNA was precipitated with ethanol. The precipitated and air-dried cDNA was resuspended in 50 μl of distilled water.

### Real-time PCR analyses

The oligonucleotide microarray data of interesting proteinases that could have a pivotal role in AIA were independently validated by real-time PCR. Cytokine expression at the mRNA level was also determined with this technique. Semiquantification of proteinase and cytokine expression by real-time PCR was performed with the Rotorgene 2000 (LTF Labortechnik, Wasserburg/Bodensee, Germany). The β-actin gene served as an endogenous control to normalize the differences in the amount of cDNA in each sample. SYBR Green I dye (Sigma) was supplied at 10,000 × concentration in dimethylsulfoxide. The enzyme Hot Star Taq (Qiagen) with the supplied reaction buffer was used for the PCR reaction. The reaction was performed in a volume of 25 μl, consisting of 2.5 μl of 10 × PCR buffer, 2.0–2.5 μl of MgCl_2 _(25 mM), 0.5 μl of dNTPs (10 mM each; Promega), 0.5 μl of Hot Star Taq (5 U/μl), 0.5 μl of primer mix (20 μM each) and 2.5 μl of 1 × SYBR Green I solution.

A standard curve was prepared by serial dilution of plasmid DNA (Vector pCR^® ^2.1-TOPO^®^; Invitrogen), containing the cDNA of the analyzed gene. The cloned fragment was identical in sequence and length with the PCR product. All samples that had to be compared for expression differences were run in the same assay as duplicates together with the standards. After completion of PCR amplification, data were analyzed with Rotorgene Software version 4.4. Data were initially expressed as a threshold cycle and are expressed as fold increases in gene expression in mice on day 0 compared with the expression on the other days investigated. The mean value of day 0 was set at 100%. After amplification was complete, the PCR products were analyzed by agarose gel electrophoresis. The primers used and the resulting PCR product sizes are given in Table 1.

### Preparation of joint extracts

Arthritic and control mice were anesthetized with ether and killed by cervical dislocation. Knee joints were dissected, skinned and snap-frozen in propane/liquid nitrogen, and stored at -80°C until further analysis. Joint extract was obtained as described by Smith-Oliver and colleagues [9]. The frozen joints were ground under liquid nitrogen with mortar and pestle. The powdered tissue was transferred to a glass homogenizer, and exactly 2 ml of sterile saline solution was added. The powder suspension was homogenized by hand for 2 min and centrifuged for 20 min at 1500 × *g *and 4°C. The supernatant was spun again for 10 min at 3000 × *g*, and the resulting supernatant was aliquoted and frozen at -70°C. Protein concentration was determined by bicinchoninic acid assay (Pierce, Rockford, IL, USA).

### Cytokine analysis in joint extracts

Concentrations of cytokines in joint extracts were determined by sandwich enzyme-linked immunosorbent assay (ELISA) in accordance with standard procedures, with the following antibody pairs: MP5-20F3 and MP5-32C11 (interleukin-6 [IL-6]; BD Biosciences, Palo Alto, CA, USA), R4-6A2 and XMG1.2 (interferon-γ [IFN-γ]; BD Biosciences), G281-2626 and MP6-XT3 (tumor necrosis factor-α [TNF-α]; BD Biosciences), MAB401 and BAF401 (IL-1β; R&D Systems, Minneapolis, MN, USA), BVD4-1D11 and BVD6-24G2 (IL-4; BD Biosciences). The second antibody of each antibody pair was biotinylated. Bound antibodies were detected with streptavidin-coupled peroxidase using *o*-phenylene diamine with 0.05% H_2_O_2 _as substrate. Optical density was measured at 492 nm in a microtiter plate-reader, model 16 598 (SLT Lab instruments, Crailsheim, Germany).

### MMP analysis in joint extracts by zymography

For analysis of proteolytic capacity, joint extracts were diluted to a final protein concentration of 1 mg/ml, mixed with sample buffer containing sodium dodecyl sulfate (SDS), glycerol and bromophenol blue. Equal amounts of each sample were separated on an SDS-polyacrylamide gel (7.5%) containing 0.8 mg/ml gelatin (Merck, Darmstadt, Germany). After SDS-polyacrylamide gel electrophoresis, the gels were washed twice with 2.5% Triton X-100 for 30 min to remove SDS, then twice with distilled water and were finally equilibrated with incubation buffer (100 mM Tris/HCl, 30 mM CaCl_2_, 0.01% NaN_3_). The gel was then incubated in incubation buffer for 20 hours at 37°C. Staining of protein was performed with Coomassie Blue solution (10 ml of acetic acid, 40 ml of distilled water, 50 ml of methanol, 0.25% Coomassie Blue G250 [SERVA, Heidelberg, Germany]) for 40 min. Destaining was performed in methanol/acetic acid/distilled water (25:7:68, by vol.). After staining, white bands on blue gels indicate enzyme species. We used human pro-MMP-2 and pro-MMP-9 as controls (Novus Molecular Inc., San Diego, CA, USA)

### Determination of MMP activity in joint extract

The synthetic peptide Mca-Pro-Leu-Gly-Leu-Dap(Dnp)-Ala-Arg-NH_2 _(no. M-1895; Bachem, Heidelberg, Germany; Mca stands for (7-methoxycoumarin-4-yl)acetic acid, Dap for L-2,3-diaminopropionic acid, and Dnp for 2,4-dinitrophenyl) was used to quantify the activity of MMPs in joint extracts. This substrate can be cleaved by different MMPs (MMP-2, MMP-3, MMP-7, MMP-8, MMP-9, MMP-10, MMP-12, MMP-13, MMP-14, MMP-17, MMP-25 and MMP-26). This fluorogenic peptide is a very sensitive substrate for continuous assays and for the *in situ *determination of matrix metalloproteinase activity. Cleavage at the Gly-Leu bond separates the highly fluorescent Mca group from the efficient Dnp quencher, resulting in an increase in fluorescence intensity.

Joint extracts were diluted with phosphate-buffered saline to give a final protein concentration of 1 mg/ml, then incubated with 5 μM substrate at 37°C in incubation buffer (100 mM Tris-HCl, 30 mM CaCl_2_, pH 7.6) for 3 hours. Active and free (not inhibited by TIMPs) MMPs cleaved the quenched substrate, which led to an increase in fluorescence at 390 nm (λ_ex _= 330 nm). The fluorescence was serially measured in black microtiter plates (Greiner, Solingen, Germany) with a fluorescence reader (FLUOstar Galaxy; BMG, B&L Systems, AS Maarssen, Netherlands). To estimate the total MMP activity, latent MMPs were activated by incubation with 2 mM aminophenylmercuric acetate (APMA; Sigma) for 15 min before the addition of substrate. The subsequent fluorescence signal after activation with APMA reflects the sum of the pro-forms of MMPs and the active forms of MMPs, which are not inhibited by TIMPs.

### Histological evaluation of paraffin sections

Mice were killed on days 0, 1, 3, 7, 14 and 21 (two animals per group) and the knees were dissected and fixed in 4% phosphate-buffered paraformaldehyde. Fixed joints were decalcified in 20% EDTA (Sigma), dehydrated and embedded in paraffin wax. Frontal sections (2 μm) of the whole knee joint were stained with safranin O (counterstained with hematoxylin) for microscopic examination. Proteoglycans of cartilage are stained red. Safranin O staining was used to reflect cartilage proteoglycan depletion during the development of experimental arthritis.

### Statistics

Analyses were performed with the statistical software SPSS/Win version 10.0 (SPSS Inc., Chicago, IL, USA). Data were analyzed with the Mann–Whitney *U*-test. After induction of arthritis, each group of animals was compared with the control day 0 group and the control normal group, respectively. For each test, *P *≤ 0.05 was considered to be statistically significant.

Correlations according to Pearson and Spearman's rho were also made with SPSS/Win version 10.0.

## Results

### Joint swelling

**Figure 1:**
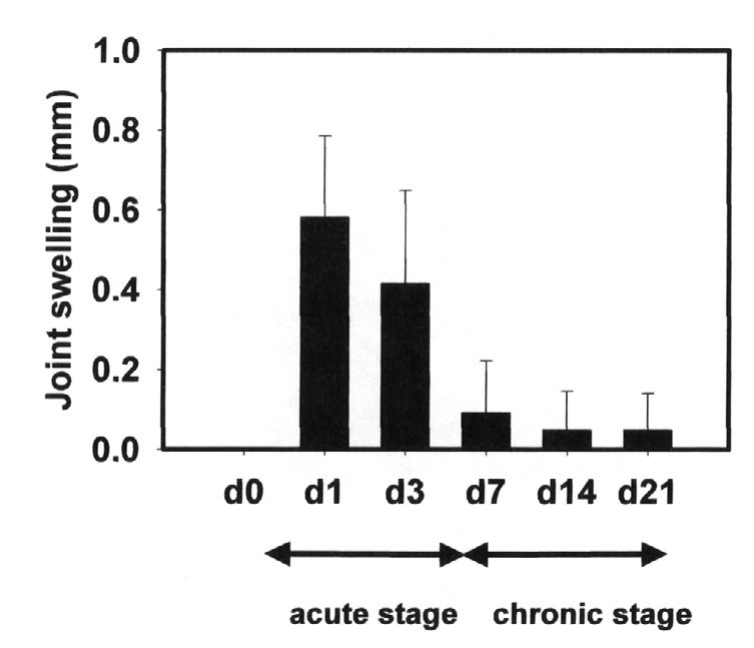
Course of joint swelling in antigen-induced arthritis. Joint swelling was expressed as the difference in diameter (mm) between the right arthritic knee joint on days 1, 3, 7, 14 and 21 and the same knee joint on day 0 before arthritis was induced. Results are expressed as means and SEM for at least five individual animals per group.

Joint swelling reached its maximum on day 1 of arthritis in the acute phase, and gradually declined until day 7 in the beginning chronic phase of AIA (Fig. 1).

### Affymetrix chip and real-time PCR analyses

**Figure 2:**
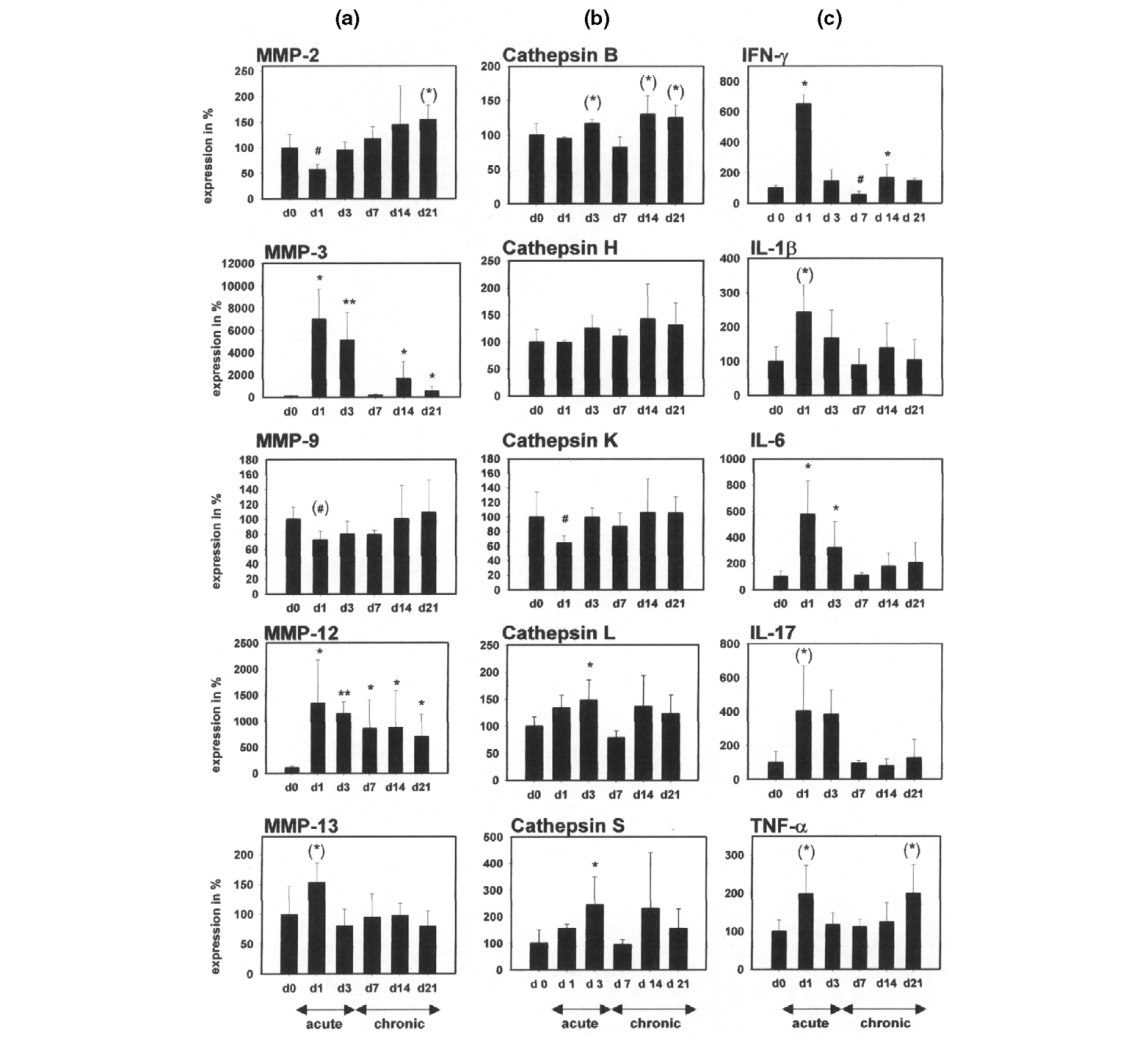
Real-time polymerase chain reaction (PCR) analyses of matrix metalloproteinase (MMP), cathepsin and pro-inflammatory cytokine expression in murine arthritic knee joints. Total RNA was isolated from murine right knee joints before (d0) and after (d1–d21) arthritis induction. mRNA samples from three (*n *= 3) (days 1, 7 and 21) or five (*n *= 5) (days 0, 3 and 14) animals were analyzed by real-time PCR with Rotorgene 2000. The mean of day 0 expression was set at 100%. The housekeeping gene β-actin was used for normalization of expression. Significant changes in comparison with day 0 expression are indicated by asterisks (increased expression) [(*) *P *≤ 0.1, * *P *≤ 0.05, ** *P *≤ 0.01] or hash signs (decreased expression) [(#) *P *≤ 0.1, # *P *≤ 0.05]. Results are expressed as means and SEM. **(a) **MMP expression; **(b) **cathepsin expression; **(c) **pro-inflammatory cytokine expression. IFN, interferon; IL, interleukin; TNF, tumor necrosis factor.

Primarily, we screened the expression patterns during the course of AIA by MG_U74Av2 Affymetrix oligonucleotide chips (array A) to investigate the role of proteinases and their inhibitors in the development of arthritis. We compared the expression at the mRNA level in arthritic knee joints of two animals on day 3 and two animals on day 14 with the expression in knee joints of two control mice on day 0. By oligonucleotide chip technology we obtained a first impression of which MMPs, TIMPs, cathepsins and cystatins were strongly and differentially expressed in murine knee joints (Table 2). We identified 5 MMPs (MMP-3, MMP-8, MMP-9, MMP-13 and MMP-14), 3 TIMPs (TIMP-1, TIMP-2 and TIMP-3), 11 cathepsins (cathepsins B, C, D, E, F, G, H, K, L, S and Z) and 3 cystatins (cystatins B, F and C) that were highly expressed at the mRNA level in murine joints during AIA and resulted in a present call as a detection signal analyzed by MAS 5.0 (Table 2). Many proteinases and inhibitors were very weakly expressed and resulted in an absent call. Among the strongly expressed proteinases and inhibitors, some MMPs, cathepsins, TIMPs and cystatins showed interesting expression patterns, which seemed to be connected with arthritis development. We used real-time PCR to validate the expression of these molecules by an independent method. We confirmed the oligonucleotide chip data for most of the proteinases investigated (except cathepsin L; Fig. 2). It was possible to investigate additional time points of AIA and to study the expression behavior more exactly by this second molecular biological method.

Of all the proteinases investigated by chip analysis and real-time PCR, the matrix metalloproteinase MMP-3 was heavily induced in the acute phase of AIA and showed the most impressive increase of expression on days 1 and 3 (Fig. 2a). Its expression level decreased in the chronic phase in comparison with the acute phase but remained significantly elevated in chronically inflamed knee joints (days 14 and 21). MMP-13 was non-significantly overexpressed on day 1 compared with day 0. No significant changes in expression of MMP-13 mRNA were found for days 3, 7, 14 and 21.

Although oligonucleotide chip technology showed MMP-12 (macrophage elastase) to be very weakly expressed (absent call; Table 2), we investigated its expression by real-time PCR, because macrophages are important in RA. MMP-12 was very strongly and significantly overexpressed in the course of AIA after arthritis induction (Fig. 2a). The significantly increased expression of MMP-12 was observed until day 21 in the chronic phase.

Gelatinase B (MMP-9) showed little change in expression at the mRNA level (Fig. 2a). The transcription was weakly but not significantly downregulated in the acute phase at the mRNA level. MMP-2 (absent call on the array) was significantly downregulated at the mRNA level on day 1 in comparison with day 0 (Fig. 2a), reaching the day 0 level again on day 3 and increasing until day 21 of the chronic phase. MMP-2 was non-significantly overexpressed at the mRNA level on day 21 in comparison with day 0.

Next we investigated in more detail the differential expression of strongly expressed cathepsins by real-time PCR. After an analysis of the array data we found some candidate genes that might have a pivotal function in AIA. We analyzed cathepsins B, H, K, L and S more thoroughly by real-time PCR. The cathepsins B and H were non-significantly overexpressed in the acute phase. The mRNAs for cathepsins L and S were significantly elevated on day 3 in murine knee joints, where arthritis was induced (Fig. 2b). In contrast to MMP-3 overexpression with the highest peak on day 1, the overexpression of cathepsins was strongest on day 3.

Because cathepsin K was strongly expressed in murine knee joints and has an essential role in joint destruction, we investigated its expression pattern by real-time PCR (Fig. 2b) and found a significant downregulation at the mRNA level on day 1 but no alteration of expression at the mRNA level on the other days in the course of AIA.

**Figure 3:**
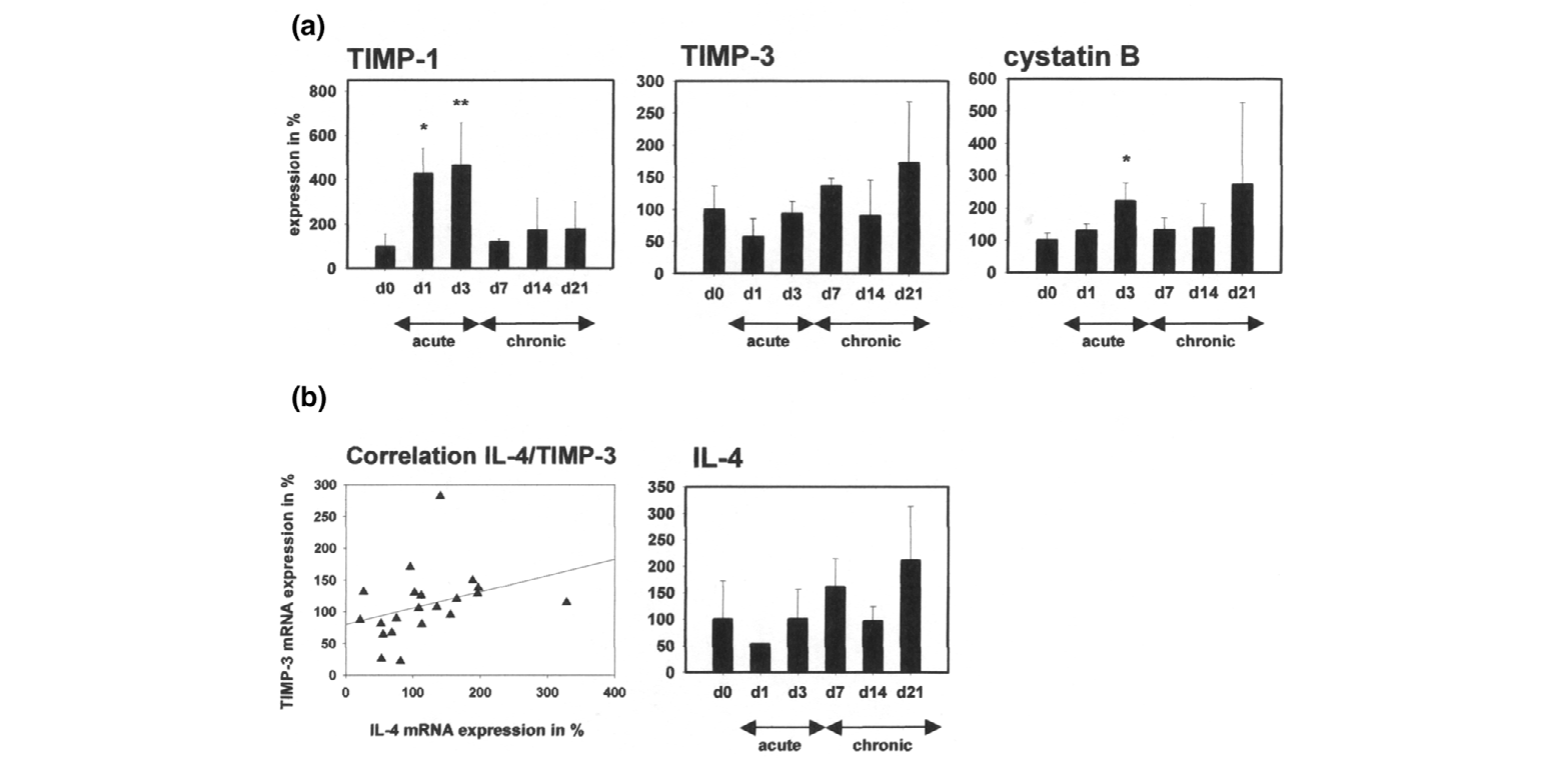
Real-time polymerase chain reaction (PCR) analyses of tissue inhibitor of metalloproteinases (TIMP)-1, TIMP-3, cystatin B and anti-inflammatory interleukin (IL)-4 expression in murine knee joints during the course of antigen-induced arthritis. Total RNA was isolated from murine right knee joints before (d0) and after (d1–d21) arthritis induction. After reverse transcription of mRNA, the expression of TIMP-1, TIMP-3, cystatin B and IL-4 was measured by real-time PCR. The housekeeping gene β-actin was used for normalization of expression. Three (*n *= 3) (days 1, 7 and 21) or five (*n *= 5) (days 0, 3 and 14) animals were analyzed by real-time PCR with Rotorgene 2000 (except IL-4, for which *n *was 3 for all investigated days). The mean of day 0 expression was set at 100%. Results are expressed as means and SEM. Significant changes in comparison with day 0 animals are indicated by asterisks (* *P *≤ 0.05, ** *P *≤ 0.01). **(a) **TIMP-1, TIMP-3 and cystatin B mRNA expression, and **(b) **anti-inflammatory IL-4 mRNA expression in knee joints significantly correlates with TIMP-3 mRNA expression according to Spearman's rho (*P *≤ 0.01).

Not only were MMPs and cathepsins differentially expressed after arthritis induction, but the expression of their inhibitors, the TIMPs and cystatins, was also changed in the course of AIA, as analyzed by Affymetrix oligonucleotide chips (Table 2). We performed real-time PCR for TIMP-1, TIMP-3 and cystatin B. TIMP-1 was the most strongly overexpressed (Fig. 3a). TIMP-3 showed a totally different expression profile from that of TIMP-1 (Fig. 3a): its expression was downregulated on day 1, increased from day 1 to day 7 and returned on day 14 to the level of control mice at day 0. The expression of cystatin B was increased about twofold on day 3 of the acute stage (Fig. 3a). Like the expression of cathepsins, the expression of cystatin B was strongest on day 3 (Fig. 3a).

### MMP measurement by fluorescence activity assay and zymography

**Figure 4:**
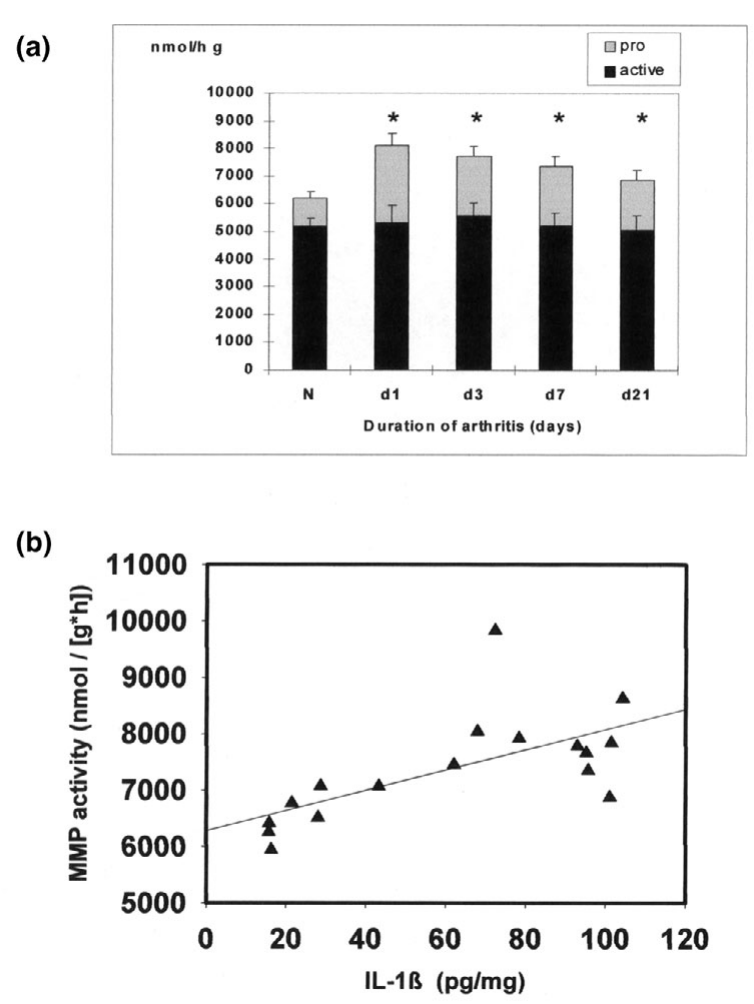
Matrix metalloproteinase (MMP) fluorescence activity assay in joint extracts at different points of time in antigen-induced arthritis (AIA). **(a) **The contents of the active forms of MMPs and the pro-forms of MMPs (after activation with aminophenylmercuric acetate) in joint extracts were measured by fluorescence assay with the synthetic peptide substrate Mca-Pro-Leu-Gly-Leu-Dap(Dnp)-Ala-Arg-NH_2_. This substrate can be cleaved by different MMPs (MMP-2, MMP-3, MMP-7, MMP-8, MMP-9, MMP-10, MMP-12, MMP-13, MMP-14, MMP-17, MMP-25 and MMP-26). An increase in the MMP content of the pro-form during the acute phase of AIA was seen. Results are expressed as means and SEM for three animals per group (*n *= 3). Significant changes in comparison with normal animals (N) are indicated by asterisks (* *P *≤ 0.05). **(b) **IL-1β protein content and total MMP activity (sum of pro-form and active form) in joint extracts is significantly correlated according to Pearson (*P *< 0.005).

Fluorescence assay data of MMP activity in arthritic knee joints showed elevated latent MMP concentrations in the acute phase of AIA, in comparison with healthy control mice (Fig. 4a). However, active MMP molecules, not bound by inhibitor molecules, were not significantly elevated in arthritic knee joints.

**Figure 5:**
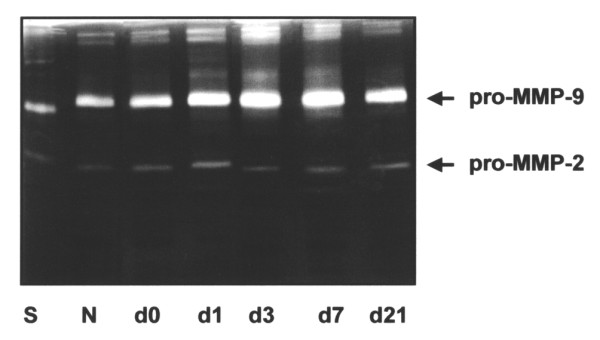
Detection, by gelatin zymography, of pro-matrix metalloproteinase-2 (pro-MMP-2) and pro-MMP-9 expression in joint extracts at different time points after the induction of antigen-induced arthritis. Joint extracts were prepared at days 0, 1, 3, 7 and 21 and from normal mice (N) (three individual animals per day). The protein concentration was determined by bicinchoninic acid protein assay, and equal amounts of protein were applied to each lane. The zymography was developed and stained as described in Materials and Methods. The picture shows a representative gel of three comparable experiments. S, standard (human pro-MMP-2, [72 kDa] and pro-MMP-9 [92 kDa]).

Zymography of total protein knee joint extracts was performed for the different time points of AIA (Fig. 5). By gelatin zymography, bands could be seen at about 105 kDa (MMP-9) and 66 kDa (MMP-2). Protein solution from arthritic knee joints showed elevated gelatinolytic activity. In contrast to mRNA studies, zymography showed an increased expression of MMP-9 (105 kDa) in the course of AIA in comparison with day 0 (Fig. 5).

### Cytokine expression during AIA

Because the production and secretion of proteinases and their inhibitors are regulated by cytokines, we investigated the expression of different cytokines at the mRNA and protein levels.

Several cytokines were differentially expressed at the mRNA level. We showed an altered expression for the pro-inflammatory cytokines IFN-γ, IL-6, TNF-α, IL-1β and IL-17 (Fig. 2c). IFN-γ was significantly elevated at the mRNA level on day 1 in the acute phase of inflammation. On day 7 it was significantly decreased at the mRNA level in comparison with day 0. IL-1β was non-significantly increased on day 1. IL-6 was significantly elevated at the mRNA level from day 1 to day 3, compared with day 0 mice at the mRNA level. Expression of IL-17 was non-significantly elevated at the mRNA level in the arthritic knee joint on days 1 and 3 of the acute phase. TNF-α expression was not significantly elevated at the mRNA level on day 1 of the acute phase or on day 21 of the chronic phase. The anti-inflammatory cytokine IL-4 was non-significantly decreased on day 1 at the mRNA level (Fig. 3b). Afterwards the mRNA expression of IL-4 increased until day 7 and returned to the starting level until day 14 of the chronic phase.

**Figure 6:**
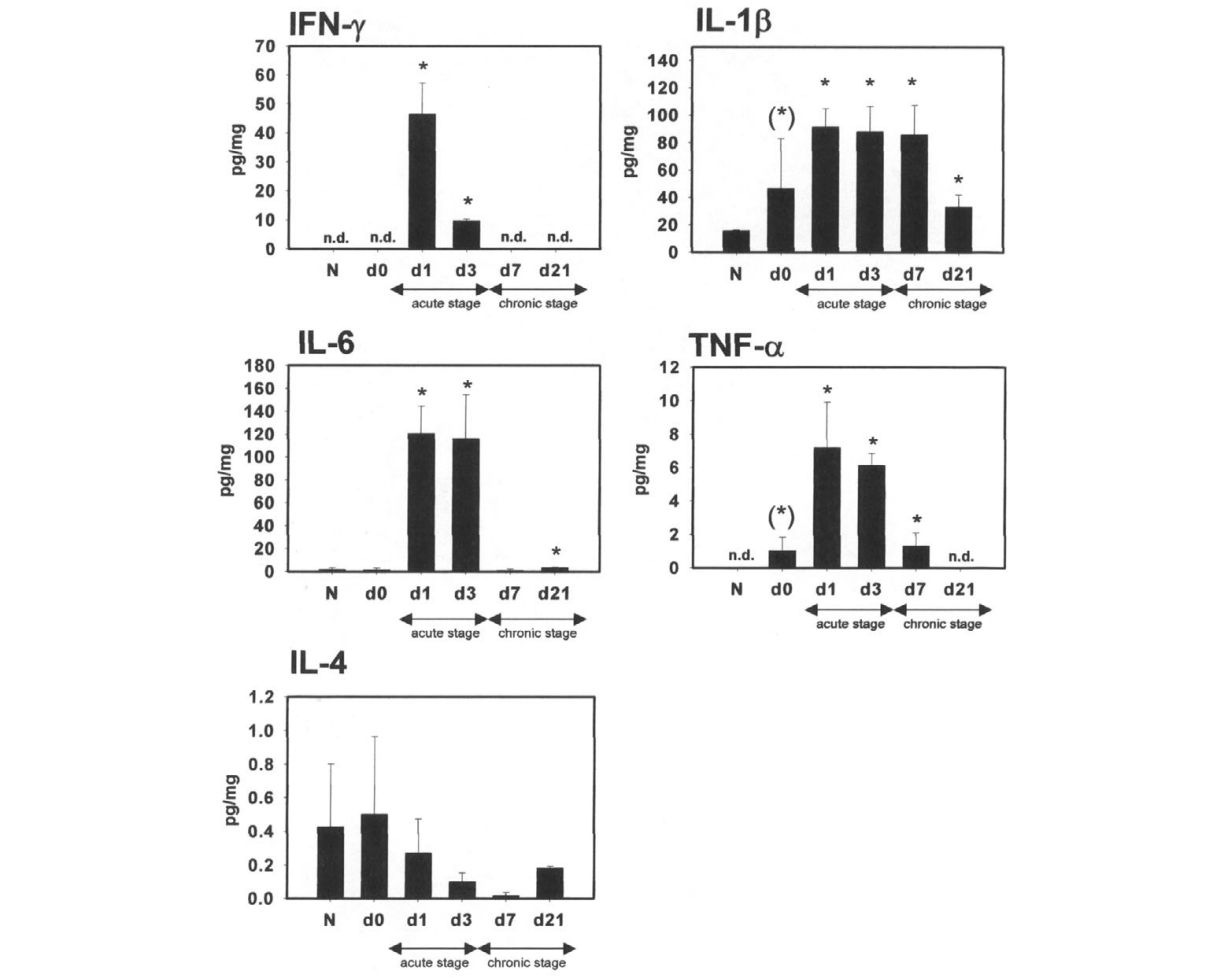
Cytokine concentrations in joint extracts at different time points after the induction of antigen-induced arthritis. The pro-inflammatory cytokines interferon (IFN)-γ, interleukin (IL)-1β, IL-6 and tumor necrosis factor (TNF)-α and the anti-inflammatory cytokine IL-4 were determined by enzyme-linked immunosorbent assay (ELISA) sandwich technique (see Materials and Methods) and were calculated in relation to the protein concentration. Results are expressed as means and SEM for at least three animals per group. Significant changes in comparison with normal animals (N) are indicated by asterisks: (*) *P *≤ 0.1, * *P *≤ 0.05, ** *P *≤ 0.01. n.d., cytokine content was not detectable by ELISA.

The expression patterns of these cytokines (exception IL-17) were also confirmed at the protein level by ELISA in murine knee joints after the induction of arthritis (Fig. 6). IFN-γ was significantly elevated at the protein level on days 1 and 3 of the acute phase. IL-1β was significantly elevated at the protein level from the induction of arthritis during the acute phase (days 1 and 3) until the beginning of the chronic phase of inflammation (day 7). IL-6 was also significantly increased in joint extracts on days 1 and 3. TNF-α expression was upregulated at the protein level on days 1 and 3 in the acute phase. In contrast to the mRNA level, no second increase in TNF-α expression was found. The anti-inflammatory cytokine IL-4 was downregulated on days 1, 3 and 7 at the protein level.

### Correlations between cytokine and proteinase expression

The expression of TIMP-3 in knee joints was significantly correlated with IL-4 expression (correlation according to Spearman's rho, *P *≤ 0.01; Fig. 3b). At the protein level, total MMP activity was significantly correlated with IL-1β protein concentration in knee joints (correlation according to Pearson *P *≤ 0.005) (Fig. 4b).

### Safranin O staining

**Figure 7:**
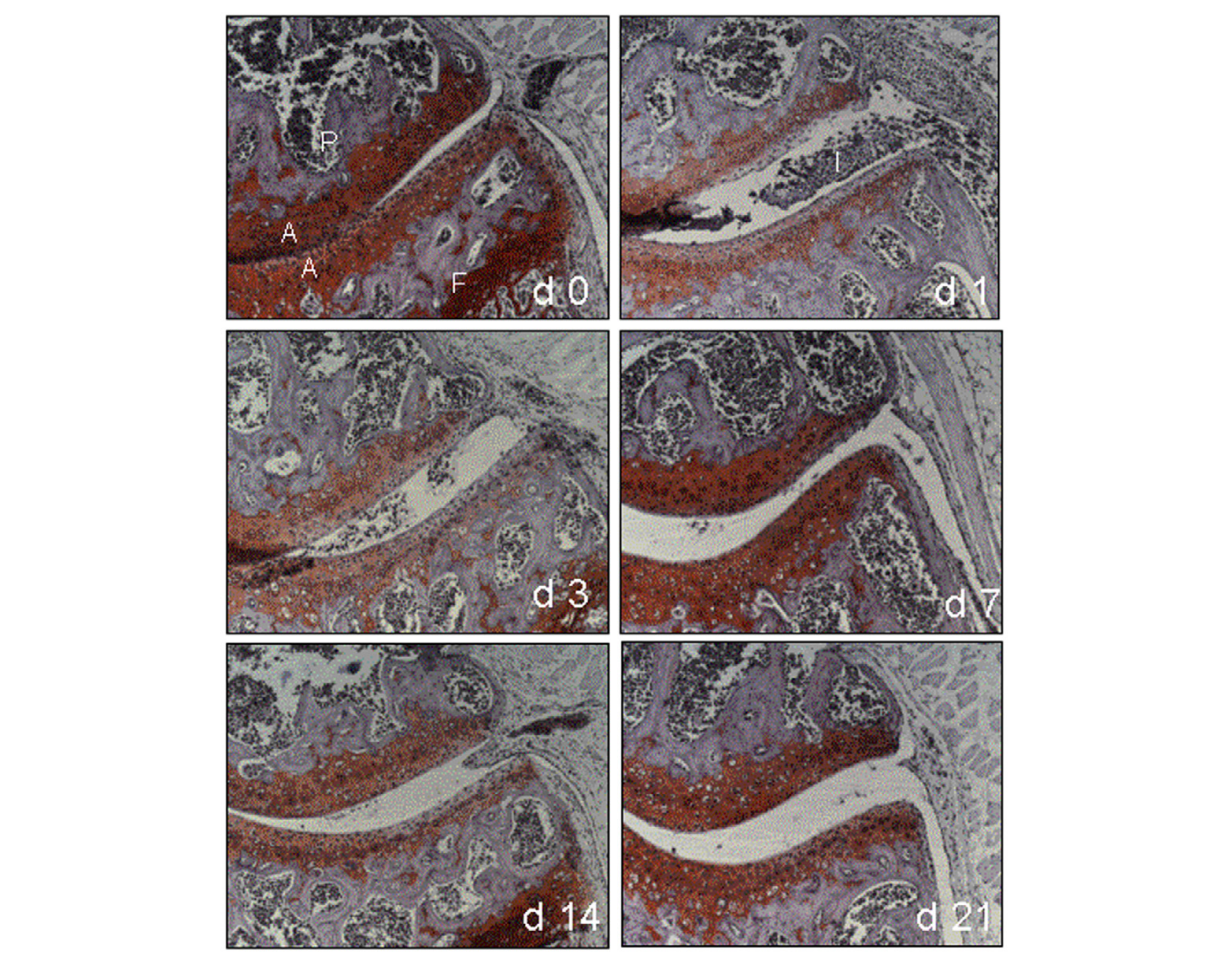
Proteoglycan (PG) degradation in articular cartilage between femur and patella. Sections were stained with safranin O and counterstained with hematoxylin (see Materials and Methods) to determine the proteoglycan contents in murine knee joints before and after the induction of antigen-induced arthritis (AIA). Proteoglycans in cartilage were stained red by safranin O. The proteoglycan content in sections before and after arthritis induction reflects the cartilage degradation during the development of AIA. A, articular cartilage; F, femur; I, infiltrate; P, patella. The proteoglycan content in articular cartilage was decreased on days 1 and 3 in comparison with control day 0 animals. Original magnification × 100.

Safranin O staining reflects the proteoglycan content of cartilage. Proteoglycan loss is an early marker of cartilage destruction. The staining intensity of proteoglycans was very weak during the acute stage of AIA on days 1 and 3, corresponding to the days of highest proteinase expression, in comparison with control joints on day 0 (Fig. 7).

## Discussion

The aim of the present study was to investigate the contribution of various proteinases to bone and cartilage degradation during the development of AIA, an experimental model of human RA.

MMPs are believed to be pivotal enzymes in the invasion of articular cartilage by synovial tissue in RA [10,11]. However, the role of individual MMPs in the pathogenesis of arthritis must be determined to identify specific targets for joint protective therapies. MMP-3 (stromelysin-1) was the proteinase that showed the highest differences in expression during the course of AIA. Our observation concurs with that of van Meurs and colleagues [12] who noted a fast upregulation of mRNA for stromelysin during acute flare-up of murine AIA. The overproduction of MMP-3 has a role not only in the direct lysis of collagen but also in the activation of other proteinases [13]. MMP-13 (collagenase 3) showed a non-significantly increased expression on day 1 of the acute phase of AIA. The significance of these results must be shown by further experimental analyses. However, Wernicke and colleagues [14] demonstrated that MMP-13 mRNA was induced at sites of cartilage erosion in synovial fibroblasts co-implanted with normal cartilage in non-obese diabetic/severe combined immunodeficient mice. In contrast to MMP-13, the macrophage elastase (MMP-12) was abundantly increased at all investigated time points in AIA. Janusz and colleagues [15] showed that murine MMP-12 degrades cartilage proteoglycan with an efficiency about equal to human MMP-12 and matrilysin (MMP-7) and twice that of stromelysin-1 (MMP-3).

The gelatinases (MMP-2/gelatinase A and MMP-9/gelatinase B) are overproduced in joints of patients with RA [16-18]. Because of their degradative effects on the extracellular matrix, the family of gelatinases has been believed to be important in progression and cartilage degradation in this disease, although their precise roles still are to be defined. Itoh and colleagues [19] showed in the model of antibody-induced arthritis that MMP-9 knockout mice displayed milder arthritis than their wild-type littermates. Surprisingly, they found that MMP-2 knockout mice display a more severe arthritis than wild-type mice. These results indicate a suppressive role of MMP-2 and a pivotal role of MMP-9 in the development of inflammatory joint disease. In contrast to most of the investigated proteinases in our study, MMP-9 showed a decreased, nearly unaffected, expression at the mRNA level. For pro-MMP-9, we detected by zymography an increased protein content in knee joints during the development of experimental arthritis. The contradiction between high MMP-9 expression at the protein level and a decreased mRNA level in our experiments can be explained by the paracrine mechanism postulated by Dreier and colleagues [20]. MMP-2 showed a significantly decreased expression on day 1. A similar regulation mechanism for MMP-2 expression as for MMP-9 has not yet been described.

Usually, metalloproteinases have been favored as potential matrix-degrading enzymes over cysteine proteinases because of an activity optimum at neutral pH. However, an increasing number of expression analyses of synovial tissues and fluids from patients with RA [21-27], and studies with models of experimental arthritis [28,29], have shown the pivotal role of cathepsins in arthritis development. Extracellular cathepsin S shows an elastin-degrading and proteoglycan-degrading activity at neutral pH [30-32]. Cathepsin B, secreted by invading tumor cells, can degrade collagen and elastin [33]. The role of cathepsin B, for instance in activation of pro-MMP-3 and its regulation in arthritis, was recently reviewed by Yan and Sloane [34]. However, the expression and function of most of the cathepsins in RA are unknown. Our results support the hypothesis that cathepsins participate in matrix-degrading processes. We detected strong increases in cathepsin S and L mRNA in the acute phase of AIA. Cathepsin B and H were non-significantly elevated on day 3 after the induction of experimental arthritis.

Current interest is focused on the role of cathepsin K in RA. Besides its expression in osteoclasts and related chondroclasts as well as in multinucleated giant cells, cathepsin K has also been described in mononuclear cells that might act as precursor cells for osteoclasts, in macrophages and in various epithelial cells. *In situ *hybridization experiments have demonstrated cathepsin K expression in synoviocytes of patients with RA at sites of synovial destruction [35,36]. The overexpression of cathepsin K as shown in RA and collagen-induced arthritis of mice could not be confirmed in AIA at the mRNA level. We found a significant decrease in the expression of cathepsin K on day 1, when IFN-γ was expressed at its highest level. A feedback mechanism, as described for MMP-9, can be excluded, because Kamolmatyakul and colleagues [37] demonstrated that IFN-γ simultaneously downregulates cathepsin K expression in osteoclasts at the protein and mRNA levels.

The cytokines, especially the pro-inflammatory cytokines such as IFN-γ, TNF-α, IL-1, IL-6 and IL-17, also have a pivotal role in the pathology of RA. They are found in large quantities in RA synovium and synovial fluid [38-42]. Furthermore, cytokine knockout and transgenic mice showed a changed susceptibility for arthritis in animal models for RA [43-46]. A variety of cytokines including IL-1, TNF-α, and IL-17 increase the production and secretion of MMPs and cathepsins in fibroblasts [21,47-49]. In our studies, the local induction of AIA led to an impressive increase in IFN-γ, TNF-α, IL-1β and IL-6 in the joints. The latent MMP concentration at the protein level significantly correlated with the IL-1β concentration in joint extracts. We obtained very precise expression profiles for the cytokines and proteinases under investigation by using real-time PCR. At the transcriptional level, the increase in many MMPs and cathepsins is correlated with the highest expression levels of pro-inflammatory cytokines on days 1 and 3. The downregulation of cathepsin K on day 1 could be another example of the fact that the time course of cytokine expression determines the time course of proteinase and inhibitor expression.

In contrast to pro-inflammatory cytokines, IL-4 is one of the major cytokines that are able to protect against severe cartilage destruction during experimental arthritis. IL-4 treatment can inhibit IL-1 mRNA levels in the synovium [50,51]. IL-4 overexpression in the arthritic knee joint strongly and locally inhibited the mRNA expression of MMP-3 in mice with collagen-induced arthritis [50]. In addition, Borghaei and colleagues [52] have shown that IL-4 suppresses the IL-1-induced transcription of collagenase 1 (MMP-1) and stromelysin-1 (MMP-3) in human synovial fibroblasts. Van Lent and colleagues [53] reported that IL-4 blocks the activation step for latent MMPs within the cartilage layer. In the course of AIA we have seen a downregulation of IL-4 on day 1 at the mRNA level, after which IL-4 mRNA expression increased until day 7. Together with the fact that IFN-γ is significantly downregulated on day 7, this might be the reason for the decreased expression of MMP-3 on day 7 at the mRNA level in our study.

The biological activity of the proteinases can be regulated transcriptionally and post-transcriptionally by different TIMPs and by activation steps by the proteolytic cleavage of latent forms. Van der Laan and colleagues [54] demonstrated that the degradation and invasion of cartilage by rheumatoid synovial fibroblasts can be inhibited by gene transfer of TIMP-1 and TIMP-3. We have demonstrated in our analyses that TIMP-1 and TIMP-3 were differentially expressed in AIA. On account of the high similarity of expression profiles, TIMP-3 transcription seems to be directly or indirectly associated with IL-4 expression. Currently, there is no experimental evidence that IL-4 upregulated the TIMP-3 mRNA expression, but a similar mechanism to the upregulation of TIMP-2 by IL-4 in dermal fibroblasts described by Ihn and colleagues [55] cannot be excluded. TIMP-1 shows an expression profile very similar to that of MMP-3, being highly overexpressed during AIA. The overexpression of TIMP-1 at the mRNA level in our analysis is smaller and appears later than MMP-3 overexpression in arthritic knee joints. Hegemann and colleagues [56] demonstrated in canine rheumatoid arthritis that the amount of TIMP-1 was not sufficient to block the increased MMP-3 activity. We showed also in murine AIA, by safranin O staining of articular cartilage, that the balance between proteinase and inhibitor expression is disturbed and results in cartilage depletion.

## Conclusion

This study was designed to detect proteinases and proteinase inhibitors that contribute to pathogenic processes in the development of experimental arthritis. We have been able to show that several MMPs, cathepsins and proteinase inhibitors are differentially expressed during the course of AIA. MMP-3, with the highest expression differences, seemed to have the major role in AIA development. We were able to show a correlation between, on the one hand, proteinase activity and proteinase inhibitor expression at the mRNA level and, on the other, cytokine expression. The mRNA data were manifested at the protein level by zymography and activity assays. Safranin O staining showed that the balance between proteinase and inhibitor expression is disturbed.

## Abbreviations

AIA = antigen-induced arthritis; APMA = aminophenylmercuric acetate; ELISA = enzyme-linked immunosorbent assay; IFN = interferon; IL = interleukin; mBSA = methylated bovine serum albumin; MMP = matrix metalloproteinases; PCR = polymerase chain reaction; RA = rheumatoid arthritis; RT = reverse transcriptase; SDS = sodium dodecyl sulfate; TIMP = tissue inhibitor of matrix metalloproteinases; TNF = tumor necrosis factor.

## Competing interests

The author(s) declare that they have no competing interests.

## Authors' contributions

US and NS performed the Affymetrix chip analysis. US, NS and CP analysed the MMP, cathepsin and cytokine expression by real-time PCR. MH performed the zymography, the MMP activity assay and the cytokine ELISAs. US wrote the manuscript. BW and RB supervised the project and gave helpful comments about the manuscript. All authors read and approved the final manuscript.

## Figures and Tables

**Table 1:** Real-time polymerase chain reaction primers for analysis of proteinase, proteinase inhibitor and cytokine mRNA expression

Gene	Forward primer	Reverse primer	Product size (base pairs)
IFN-γ	5'-gcg tca ttg aat cac acc tg-3'	5'-gac ctg tgg gtt gtt gac ct-3'	104
IL-1β	5'-cag gca ggc agt atc act ca-3'	5'-atg agt cac aga gga tgg gc-3'	140
IL-4	5'-agc tgc aga gac tct ttc gg-3'	5'-tgc tct tta ggc ttt cca gg-3'	111
IL-6	5'-ccg gag agg aga ctt cac ag-3'	5'-cag aat tgc cat tgc aca ac-3'	134
IL-17	5'-cct aag aaa ccc cca cgt tt-3'	5'-ttc ttt tca ttg tgg agg gc-3'	129
TNF-α	5'-acg gca tgg atc tca aag ac-3'	5'-gtg ggt gag gag cac gta gt-3'	116
MMP-2	5'-agc gtg aag ttt gga agc at-3'	5'-cac atc ctt cac ctg gtg tg-3'	105
MMP-3	5'-tgg aga tgc tca ctt tga cg-3'	5'-gcc ttg gct gag tgg tag ag-3'	120
MMP-9	5'-cat tcg cgt gga taa gga gt-3'	5'-att ttg gaa act cac acg cc-3'	118
MMP-12	5'-ttt gga gct cac gga gac tt-3'	5'-cac gtt tct gcc tca tca aa-3'	116
MMP-13	5'-agt tga cag gct ccg aga aa-3'	5'-ggc act cca cat ctt ggt tt-3'	105
TIMP-1	5'-ggt gtt tcc ctg ttt atc-3'	5'-tag ttc ttt att tca cca tct-3'	254
TIMP-3	5'-ttg ggt acc ctg gct atc ag-3'	5'-agg tct ggg ttc agg gat ct-3'	132
Cathepsin B	5'-gga gat act ccc agg tgc aa-3'	5'-ctg cca tga tct cct tca ca-3'	121
Cathepsin H	5'-gg cag agc ctc aga att gc-3'	5'-act ggc gaa aca aca ttt gc-3'	109
Cathepsin K	5'-ggg cca gga tga aag ttg ta-3'	5'-cac tgc tct ctt cag ggc tt-3'	106
Cathepsin L	5'-atc ccc aag tct gtg gac tg-3'	5'-tca gtg aga tca gtt tgc cg-3'	145
Cathepsin S	5'-aga gaa ggg ctg cgt cac t-3'	5'-gat atc agc ttc ccc gtt ttc ag-3'	117
Cystatin B	5'-tgc tga caa ggt cag acc ac-3'	5'-gca acc acg tcc tac att ca-3'	133
β-actin	5'-cca cag ctg aga ggg aaa tc-3'	5'-tct cca ggg agg aag agg at-3'	108

IFN, interferon; IL, interleukin; MMP, matrix metalloproteinase; TIMP, tissue inhibitor of metalloproteinases; TNF, tumor necrosis factor.

**Table 2:** Results of oligonucleotide chip analyses for matrix metalloproteinase (MMP), cathepsin, tissue inhibitor of metalloproteinases (TIMP) and cystatin expression

Gene	Probe set ID	First screening set						Second screening set					
		Day 0		Day 3		Day 14		Day 0		Day 3		Day 14	
		S	D	S	D	S	D	S	D	S	D	S	D
MMP-2	161509_at	34	A	17,2	A	6,2	A	14,5	A	19,2	A	31,8	A
MMP-2	95663_at	22,8	A	23,6	A	12,1	A	25,6	A	33,9	A	22,8	A
**MMP-3**	**98833_at**	**35,6**	**P**	**97,4**	**P**	**38,5**	**P**	**35**	**P**	**424,5**	**P**	**84,3**	**P**
MMP-7	162318_r_at	24,2	A	4	A	3,9	A	3,8	A	11,7	A	2,2	A
MMP-7	92917_at	3	A	5,4	A	2,6	A	4,1	A	1,8	A	2,7	A
**MMP-8**	**94769_at**	**940,1**	**P**	**830,2**	**P**	**823,8**	**P**	**998,7**	**P**	**778,2**	**P**	**1006,5**	**P**
**MMP-9**	**162369_f_at**	**237**	**P**	**170,6**	**P**	**175,5**	**P**	**172,1**	**P**	**179,4**	**P**	**147,1**	**P**
**MMP-9**	**99957_at**	**2481,1**	**P**	**2287,7**	**P**	**1993,7**	**P**	**1838,9**	**P**	**2177**	**P**	**2264,3**	**P**
MMP-10	94724_at	16	A	1,4	A	2	A	2	A	5,5	A	0,9	A
MMP-11	100016_at	42,3	A	47,7	M	26,1	A	40,1	A	30,5	A	53	A
MMP-12	95338_s_at	20,1	A	38,1	A	7	A	19,7	A	21,2	A	8,1	A
MMP-12	95339_r_at	33	A	81,9	A	32,6	A	15,2	A	71,8	A	30	A
MMP-13	161219_r_at	64,5	P	61,4	M	62,9	A	56,6	A	69,8	P	35,1	A
**MMP-13**	**100484_at**	**1202,4**	**P**	**927**	**P**	**843,5**	**P**	**1510,5**	**P**	**1373,3**	**P**	**1184,2**	**P**
**MMP-14**	**160118_at**	**796,5**	**P**	**876,4**	**P**	**717,9**	**P**	**607,6**	**P**	**611,8**	**P**	**753,3**	**P**
MMP-15	93612_at	40,7	A	9,4	A	7,3	A	24,6	A	11,5	A	15,7	A
MMP-16	98280_at	12,7	A	3,7	A	5,3	A	8,6	A	8,8	A	5,5	A
MMP-17	92461_at	35,4	A	43,4	M	26,3	A	35,4	A	56,1	A	43	A
MMP-24	160665_at	4,4	A	3,1	A	2,5	A	5,2	A	4,4	A	3,8	A
**TIMP-1**	**101464_at**	**525,5**	**P**	**617,2**	**P**	**427,7**	**P**	**383,4**	**P**	**652,7**	**P**	**407,3**	**P**
**TIMP-2**	**93507_at**	**147,6**	**P**	**122,9**	**A**	**126,3**	**A**	**152,4**	**P**	**188,1**	**P**	**131,3**	**A**
**TIMP-3**	**160519_at**	**367,3**	**P**	**451,3**	**P**	**484,6**	**P**	**553,2**	**P**	**518,8**	**P**	**521**	**P**
**Cathepsin B**	**92256_at**	**78,3**	**P**	**113,1**	**P**	**46,5**	**P**	**102,5**	**P**	**104,8**	**P**	**88,9**	**P**
**Cathepsin B**	**94831_at**	**2150,4**	**P**	**2480,2**	**P**	**2244**	**P**	**1666,8**	**P**	**2013**	**P**	**1694**	**P**
Cathepsin B	95608_at	119,6	P	96	M	117,5	P	156,3	P	121,7	P	100,7	P
**Cathepsin C**	**101019_at**	**286,7**	**P**	**188,5**	**P**	**192,6**	**P**	**298,7**	**P**	**339,5**	**P**	**197,6**	**P**
**Cathepsin C**	**101020_at**	**145,8**	**P**	**97**	**P**	**101,6**	**P**	**150,6**	**P**	**251,5**	**P**	**126**	**P**
Cathepsin C	161251_f_at	42,8	P	53,2	A	42,8	M	64,2	A	52,2	A	68,8	A
**Cathepsin D**	**93810_at**	**1934**	**P**	**2280,4**	**P**	**1968,3**	**P**	**1551,4**	**P**	**1526**	**P**	**1994,3**	**P**
**Cathepsin E**	**104696_at**	**853,1**	**P**	**1383,5**	**P**	**1680,7**	**P**	**1619,9**	**P**	**1367,1**	**P**	**1368,3**	**P**
**Cathepsin F**	**97336_at**	**169**	**P**	**196,9**	**P**	**186,6**	**P**	**149,5**	**P**	**136**	**P**	**181,7**	**P**
**Cathepsin G**	**92924_at**	**1329**	**P**	**931**	**P**	**952,8**	**P**	**813,1**	**P**	**860,2**	**P**	**910,6**	**P**
**Cathepsin H**	**94834_at**	**506,5**	**P**	**565,4**	**P**	**399**	**P**	**319,9**	**P**	**399,2**	**P**	**398,2**	**P**
**Cathepsin K**	**160406_at**	**1312,8**	**P**	**1256,8**	**P**	**1296,1**	**P**	**1048,2**	**P**	**971,3**	**P**	**1202,5**	**P**
**Cathepsin L**	**101963_at**	**327,5**	**P**	**237,1**	**P**	**251,3**	**P**	**385,8**	**P**	**359,1**	**P**	**308,2**	**P**
**Cathepsin S**	**98543_at**	**1184,2**	**P**	**2003,7**	**P**	**1208,8**	**P**	**1549,8**	**P**	**2321,3**	**P**	**1349,6**	**P**
Cathepsin W	92214_at	11,6	A	7,4	A	23,1	A	38,7	A	17,8	A	44	A
**Cathepsin Z**	**92633_at**	**273,1**	**P**	**278,4**	**P**	**215,8**	**P**	**274,8**	**P**	**228**	**P**	**265,3**	**P**
Cathepsin 7	97678_r_at	0,5	A	0,4	A	2,6	A	10,8	A	4	A	1	A
**Cystatin B**	**100581_at**	**895,4**	**P**	**964,3**	**P**	**881,6**	**P**	**896,3**	**P**	**1017,8**	**P**	**760,4**	**P**
**Cystatin F**	**102638_at**	**360,4**	**P**	**375,4**	**P**	**280,4**	**P**	**343**	**P**	**334,3**	**P**	**366,8**	**P**
Cystatin 8	103245_at	11,7	A	24,7	P	8,6	A	10,1	A	10,8	A	11,3	A
Cystatin 9	103296_at	5,2	A	12,8	A	6,4	A	25,2	A	2,5	A	32,4	A
**Cystatin C**	**161522_i_at**	**130,6**	**P**	**180,2**	**P**	**201,9**	**P**	**105,7**	**P**	**91,1**	**P**	**215,5**	**P**
**Cystatin C**	**99586_at**	**2978,9**	**P**	**2793,1**	**P**	**3150,2**	**P**	**2467,9**	**P**	**2313,2**	**P**	**2982,7**	**P**
**β-actin**	**M12481_5_at**	**9831**	**P**	**7177,8**	**P**	**7669,6**	**P**	**9942,5**	**P**	**9216,7**	**P**	**16027,1**	**P**
**β-actin**	**M12481_M_at**	**10941**	**P**	**11693**	**P**	**10336,4**	**P**	**11128**	**P**	**11603,2**	**P**	**19708,7**	**P**
**β-actin**	**M12481_3_at**	**12579**	**P**	**15514**	**P**	**12970,5**	**P**	**14876**	**P**	**12678,2**	**P**	**21765,3**	**P**

The MG_U74Av2 Affymetrix oligonucleotide chip (array A) was used to analyze gene expression in murine arthritic knee joints during the course of antigen-induced arthritis (see Materials and Methods). We hybridized six chips with samples from two individual animals for each point of time (first screening set, days 0, 3 and 14; second screening set, days 0, 3 and 14). The table contains the following information: analyzed genes with Affymetrix probe set IDs, signal intensity (S) and detection information (D), with an absent call indicated by A, a marginal call by M and a present call by P. Probe sets with a present call (P) for all investigated days are indicated in bold.
